# Multimodality imaging of cardiac B-cell lymphoma

**DOI:** 10.1007/s12350-022-02904-x

**Published:** 2022-01-26

**Authors:** Martina Boscolo Berto, Giancarlo Spano, Daniel Rhyner, Adrian T. Huber, Christoph Gräni

**Affiliations:** 1 Department of Cardiology, Inselspital, Noninvasive Cardiac Imaging, Bern University Hospital, University of Bern, Freiburgstrasse 4, 3010 Bern, Switzerland; 2 Department of Diagnostic, Interventional and Paediatric Radiology, Inselspital, Bern University Hospital, University of Bern, Bern, Switzerland

## Introduction

We illustrate how multimodality imaging using cardiac magnetic resonance (CMR) and 18F-fluodeoxyglucose (^18^F-FDG) Positron emission tomography/computed tomography imaging (PET/CT) can help diagnose a cardiac B-cell lymphoma and document treatment success.

## Case Presentation

**Figure 1:**
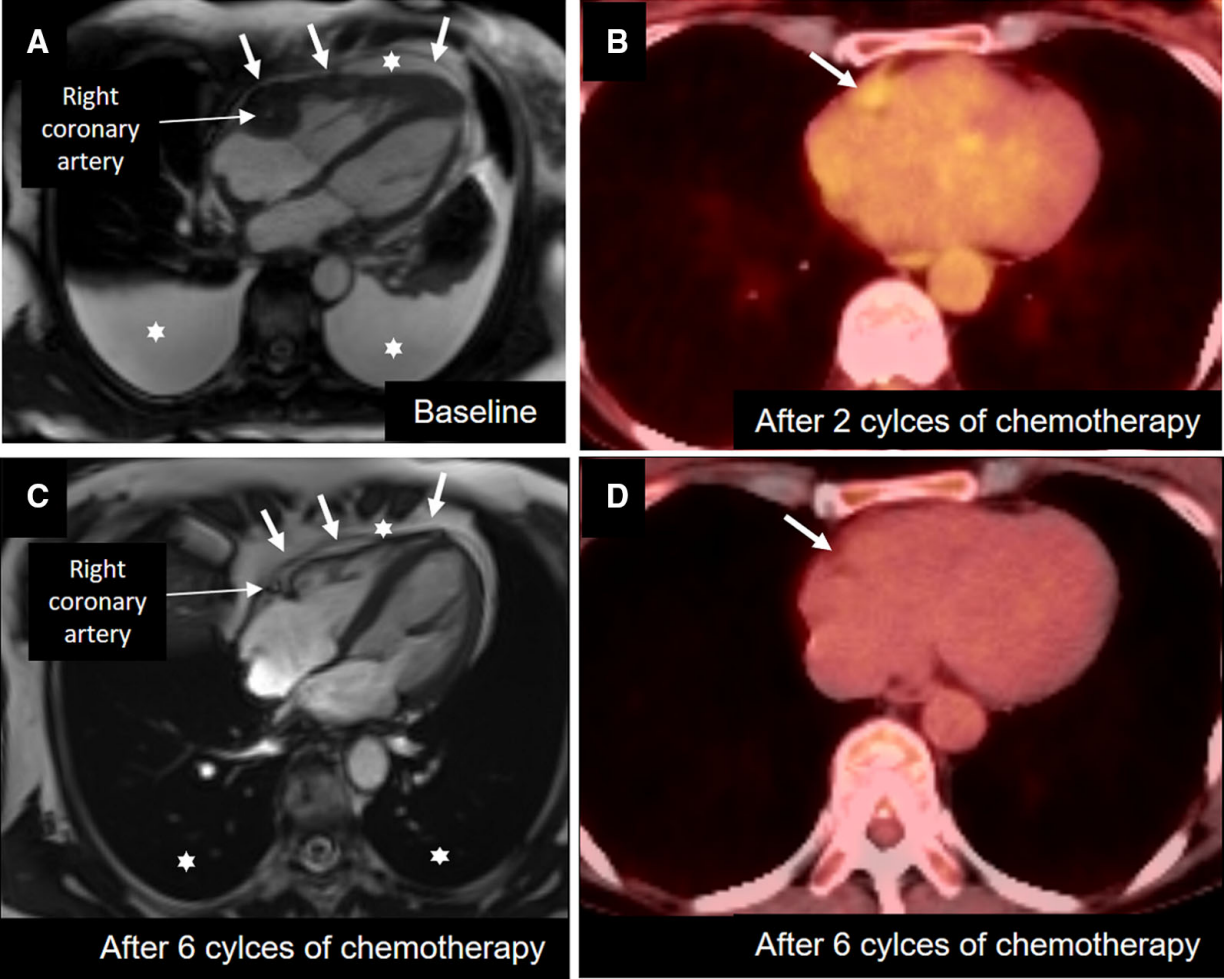
Baseline and follow-up CMR and 18F-FDG PET/CT imaging. **A** Steady-state free precession-cine imaging shows a defined, heterogeneous myocardial mass (arrows) involving the right atrial ventricular groove, surrounding the right coronary artery and extending to the right ventricle wall, as well as moderate pericardial and pleural effusion (asterisks). CMR is the ideal modality to differentiate angiosarcoma (i.e., typically involving the right atrial appendage and not surrounding the right coronary artery, central liquefaction necrosis with rim enhancement with lack of central enhancement) from lymphoma like in our case with typically diffusely involvement of the right atrioventricular groove and completely surrounding the right coronary artery, extending towards the right ventricle and mostly homogenous perfusion and heterogenous late gadolinium enhancement. **B** PET/CT shows little ^18^F-FDG uptake after 2 cycles. **C** and **D** CMR and ^18^F-FDG showed complete remission after 6 cycles of chemotherapy

**Figure 2:**
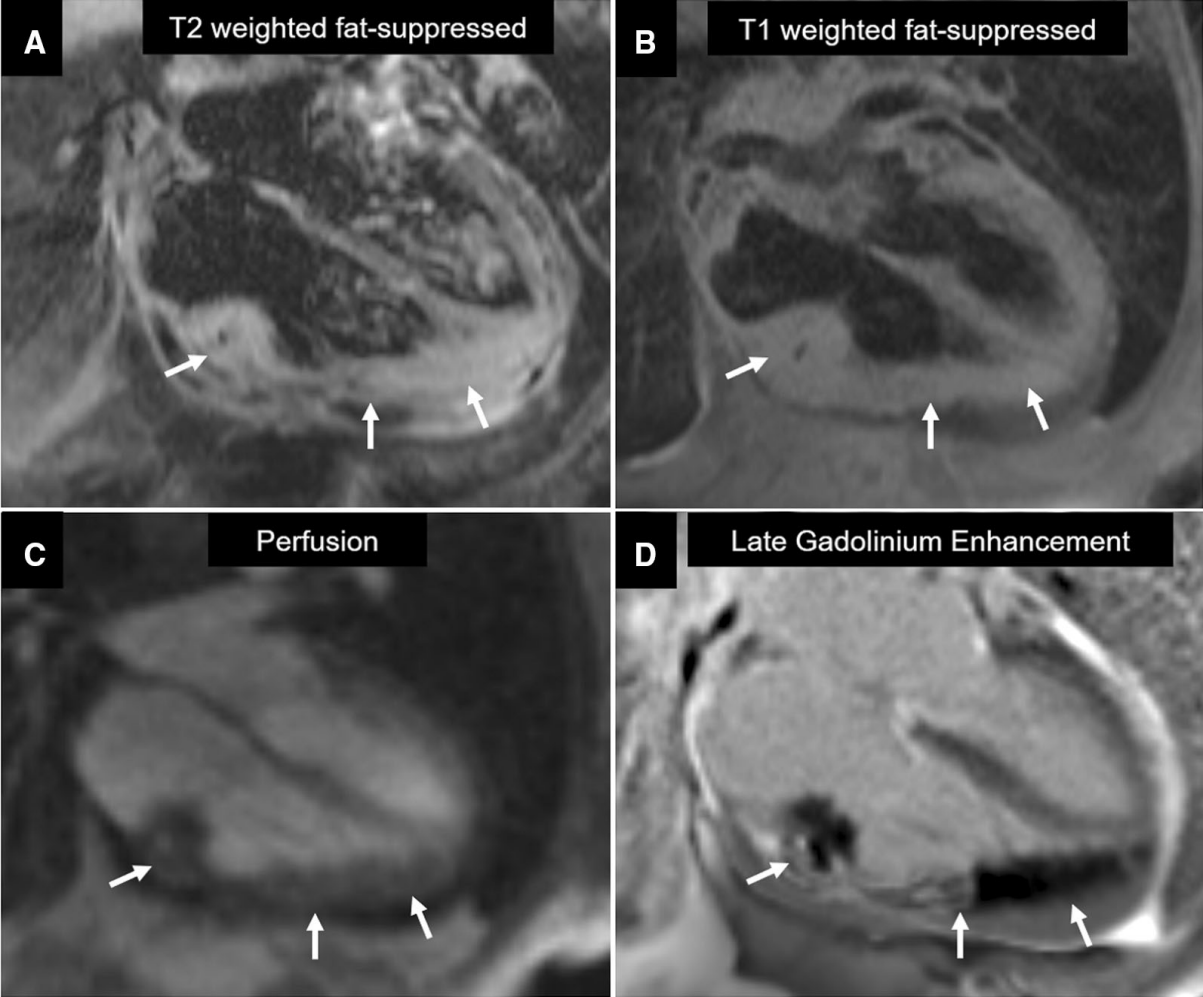
Baseline CMR tissue characterization and perfusion of the mass. **A** T2-weighted fast-suppressed imaging showing hyperenhancement of the mass, compatible with edema. **B** T1-weighted fat-suppressed sequence showing isointensity of the heterogeneous mass. **C** First-pass perfusion images show diffuse but homogeneous enhancement of the tumor. **D** T1-weighted inversion recovery showing heterogeneous late gadolinium enhancement of the mass

A 65-year-old woman presented to the emergency department with a 6-month history of progressive dyspnea, intermitted tachycardia, and recent weight loss. A computed tomography showed no lung embolism and no lung or abdominal tumor; however, pericardial and pleural effusion was depicted and the suspicion of a cardiac tumor in the right ventricular groove was raised. Besides confirmation of pericardial and pleural effusion (Figure 1), CMR depicted a T1 hypointense, T2 hyperintense, perfused, and heterogeneous late gadolinium enhancing mass (Figure 2) within the right atrioventricular groove, surrounding the right coronary artery and infiltrating the free wall of the right ventricle. No other primary tumor or metastasis could be found. A primary malignant cardiac lymphoma was suspected and the cytological analysis of the pericardial effusion confirmed a cardiac B-cell lymphoma. After 2 cycles of chemotherapy with rituximab cyclophosphamide–doxorubicin–vincristine–prednisone, PET/CT showed a decreased mass in size with only little ^18^F-FDG uptake within the same location. After completing of the entire 6 cycles of chemotherapy, CMR and ^18^F-FDG PET/CT revealed a complete remission with resolution of the symptoms.

## Discussion

Primary malignant cardiac tumors are extremely rare and establishing the diagnosis may be challenging. Angiosarcoma and lymphoma are two most common malignant primary cardiac tumors and are usually located in the right heart. CMR is the ideal modality to differentiate angiosarcoma from lymphoma.^1^ ^18^F-FDG PET/CT is helpful to differentiate a malignant from a benign cardiac tumor and to document treatment success.^2,3^ Multimodality imaging is crucial for early noninvasive assessment of primary cardiac tumors, helps guiding further investigations, treatment decision and allows documentation of therapeutic success.
